# Shade Increased Seed Yield and Quality of *Incarvillea sinensis* var. *przewalskii*

**DOI:** 10.3390/plants12162934

**Published:** 2023-08-14

**Authors:** Yan Wang, Jingjing Wang, Dali Chen, Zhenning Hui, Xiaowen Hu

**Affiliations:** 1 State Key Laboratory of Herbage Improvement and Grassland Agro-Ecosystems, Lanzhou 730000, China; wangy19@lzu.edu.cn (Y.W.); chendl16@lzu.edu.cn (D.C.); huizhn20@lzu.edu.cn (Z.H.); 2 Key Laboratory of Grassland Livestock Industry Innovation, Ministry of Agriculture and Rural Affairs, Lanzhou 730000, China; 3 Engineering Research Center of Grassland Industry, Ministry of Education, Lanzhou 730000, China; 4 College of Pastoral Agriculture Science and Technology, Lanzhou University, Lanzhou 730000, China

**Keywords:** shade treatment, seed development, reproductive branches position, high-quality seeds, breeding program

## Abstract

*Incarvillea sinensis* var. *przewalskii* has attracted great attention because of the anticancer value of its alkaloids and the potential use of the species for ecological restoration. However, the scarcity of high-quality seeds has significantly hindered the cultivation and efficient utilization of this species. Understanding how seeds respond to maternal environmental conditions is crucial for developing high-yield and top-notch seed accessions, but the available knowledge in this area is limited. Here, we determined the effect of shading treatments on seed development, seed quality, and yield. Compared to the control, shade significantly increased the seed germination rate and 1000-seed weight by 29.2% and 25.6%, respectively. Regardless of light conditions, the seed germination rate and 1000-seed weight decreased by 7.13% and 37.5%, respectively, as the fruit positioned from base to apical. The seed yield per plant was 27.9% higher under shade than under the control treatment. The structural equation model showed that shade promoted seed yield through increasing flowers per reproductive branch and seed numbers per capsule. These findings suggest that adjusting shading conditions and optimizing inflorescence development can lead to high-yield and high-quality seeds. Additionally, prioritizing the number of flowers per reproductive branch and seeds per capsule in breeding programs can further enhance the seed yield of *I*. *sinensis* var. *przewalskii*.

## 1. Introduction

*Incarvillea sinensis* var. *przewalskii* is considered an important perennial species of Bignoniaceae with excellent ornamental and medicinal values [1,2,3]. It is distributed in southwestern, northwestern to northeastern China under mingled forest (2000–2600 m above sea level) [1]. Recently, *I. sinensis* var. *przewalskii* gained attention because of the anticancer value of the alkaloids (incarvillateine and acteoside) extracted from the whole plant [3,4]. In addition, it holds potential for the ecological restoration of degraded grasslands and serves as an ornamental species for landscaping because of its wide distribution, strong adaptability, abundant and long-flowering flowers, and a well-developed root system that benefits soil fixation, ecological stability, and biodiversity preservation. Despite generally high seed production in the wild for *I. sinensis* var. *przewalskii*, issues such as seed dehiscence, seed shattering during middle and late seed maturity stages, seed dormancy, and low seed germination have greatly hindered its cultivation and utilization. Consequently, improving seed yield and quality for artificial cultivation has become a new focus for *I. sinensis* var. *przewalskii.*

The seed yield of crops is mainly determined by the accumulation and distribution of photosynthetic products after anthesis [5,6,7]. Generally, *I. sinensis* var. *przewalskii* grows on valley bottoms and under mingled forest in arid habitats, often in association with other plants [4], resulting in plant growth under different degrees of shading. Excessive light exposure can lead to the overgeneration of reactive oxygen species [8], restricting photosynthesis. Planting under shade conditions could effectively mitigate the adverse effects of excessive light on the plant metabolism, quality, and growth [9]. Some studies have shown that appropriate shading significantly increases the biomass accumulation of seedlings and flower bud differentiation and promotes seed yield [10,11,12]. In contrast, the negative effects of shade on seed yield have also been reported [13,14,15,16,17]. Furthermore, environmental conditions during the reproductive period, particularly the intensity and quality of the solar radiation intercepted by the canopy, play a crucial role in determining crop yield and yield components [18,19]. Insufficient sunlight reduces photosynthetic activity and assimilation, resulting in flower abscission [20]. Light enrichment initiated at late vegetative or early flowering stages increased soybean plentiful pod number, resulting in seed yield increased [21]. Seed quality is mainly determined by germination, seed mass, and its intrinsic quality, which are usually affected by the environmental factors experienced by maternal plants, especially light intensity [22]. Light shading has been found to significantly improve the seed volume, weight, and nutritional quality parameters of oilseed peony during the slow growth and the maturation periods of seed development [22], while a decrease in both light intensity and quality led to a decrease in the seed germination rate of *P. vulgaris* [23]. However, the knowledge of shading on seed development and consequent seed yield and quality in *I. sinensis* var. *przewalskii* is limited.

*I. sinensis* var. *przewalskii* has racemose inflorescences, and our previous study found that its flowering period is from June to September, with each reproductive branch capable of bearing 5–15 flowers. This implies that seeds located at different positions in the reproductive branch experience varying environmental and physiological conditions. For example, seeds located at the basal are closer to photosynthetic organs, mature earlier, but receive less light than the apical. The differences in light perception owing to different maturation times, allocation of resources to different positions, and distance from photosynthetic organs can result in a variation in seed quality and yield [11,15,24,25,26]. Koller found seeds germinating at different positions on the reproductive branch of *Atriplex dimorphostegia*, with seeds germinating earlier and at a higher rate at the base of the reproductive branch than seeds located distal to the reproductive branch [10,27]. In addition, adjusting planting density, extending the seed-filling period, and reducing unproductive side branch growth during flowering have been found to increase assimilate availability [28]. Segregating the seeds of the apical portion from the basal portion at harvest is an important measure to optimize crop growth and to achieve maximum biomass and seed yield and quality [29,30,31]. Studies have shown that narrow rows or increased seeding rates made higher densities possible, thereby increasing soybean yield [32], and removing winter oilseed rape pods of side branches at flowering improved seed weight [33]. These studies suggest that seed development and location on the maternal plant play a key role in fruit size, seed production, and quality, and it is possible to apply field management practices to improve seed quality and yield. Therefore, clarifying the interaction between shading and seed positions, especially the possible impact on medicinal plants’ growth and quality, needs more attention.

As the reproductive organ of plants, seeds are a prerequisite for their growth and development and play a crucial role in the life cycle of plants. Determining the dynamics of seeds and capsule development, as well as seed quality and yield under shade treatments is an important part of the production process and holds great significance for the conservation, utilization, and promotion of the plant. Therefore, we carried out a series of works under shade conditions in the field and explored the following issues. (i) What are the effects of shade treatments on seed quality and yield? (ii) What is the difference in the seed quality of capsules at different positions on the reproductive branches under shade conditions? (iii) How does shade affect the relationship between seed yield components and yield?

## 2. Results

### 2.1. Effects of Shade on Seed Development

**Figure 1:**
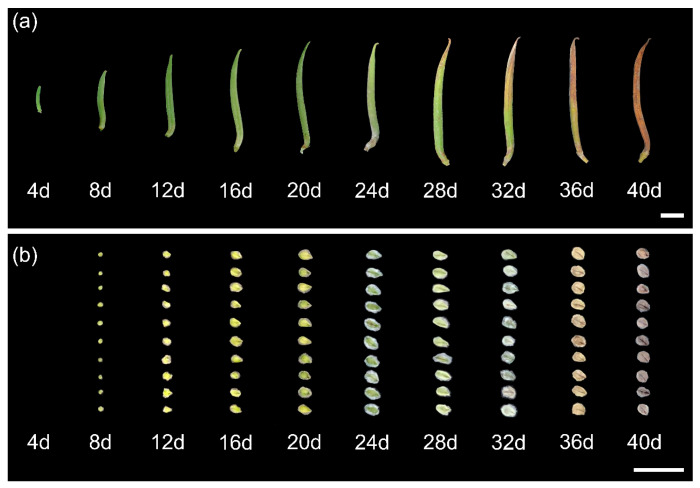
The development progress of (**a**) capsule and (**b**) seed under shading treatment. Scale bar = 1 cm.

**Figure 2:**
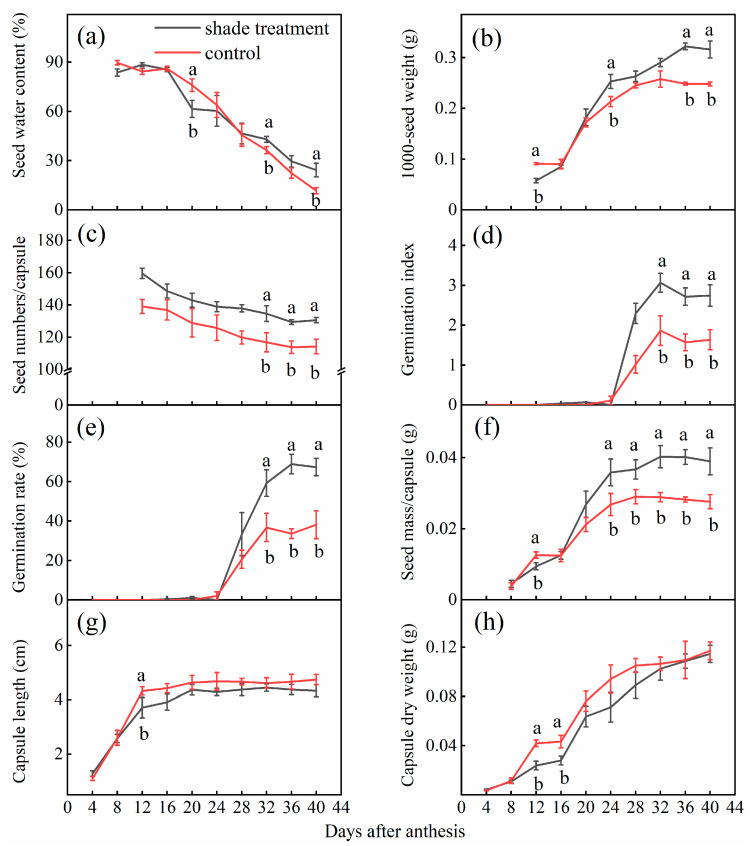
Effect of shade on the development of seed and capsule traits. (**a**) Seed water content. (**b**) 1000-seed weight. (**c**) Seed numbers per capsule. (**d**) Germination index. (**e**) Germination rate. (**f**) Seed mass per capsule. (**g**) Capsule length. (**h**) Capsule dry weight. Different lowercase letters indicate a significant difference between shade and control (*p* < 0.05). The red line represents the control treatment and the black line represents the shading treatment.

Regardless of the shade or control treatment, 1000-seed weight, germination index, germination rate, seed mass per capsule, capsule length and dry weight tended to increase and then remain constant with increasing development time, while the seed water content and seed numbers per capsule tended to decrease and then reach a stable state (Figure 1 and Figure 2). From 4 to 24 days after anthesis, the capsule developed rapidly, and the capsule length, dry weight, seed mass per capsule, and 1000-seed weight increased by 2.3, 16.6, 5.7, and 2.8 times, respectively (Figure 2b,f–h). From 37 to 40 days, both seed and capsule traits did not change a lot.

Shade significantly affected capsule and seed development (Figure 1a,b and Figure 2, Table 1). The shade, days after anthesis (DAA), and their interaction had significant effects on all seed and capsule traits, except for the effect of the interaction on seed water content (*p* = 0.08), seed numbers per capsule (*p* = 0.93), capsule length, and dry weight (*p* = 0.98, *p* = 0.99) (Table 1). Shading treatments did not affect the maturity date as 1000-seed weight, germination percentage, seed mass per capsule, seed length, and capsule dry weight reached their maximum at 32–40 DAA under both shade treatment and control conditions (Figure 2b,e–h). Shade significantly inhibited capsule length and dry weight in the early stages, while there were no significant differences between the control and shade during the late development stage (*p* > 0.05) (Figure 2g,h). Additionally, compared to the control, germination percentage, 1000-seed weight, seed numbers per capsule, and seed mass per capsule under shade treatment at late seed maturity increased by 29%, 25.7%, 14.4%, and 50.3%, respectively. Compared to the control, shade significantly increased capsule length and capsule dry weight by 13.0% and 55.6% at the early stage of development (before 16 DAA), respectively, and there was no significant difference between the two treatments after maturity. Seed water content was about 20% for both control and shade treatment at late seed maturity (40 DAA) (Figure 2a).

### 2.2. Effects of Shade and Capsule Position on Seed Quality

**Figure 3:**
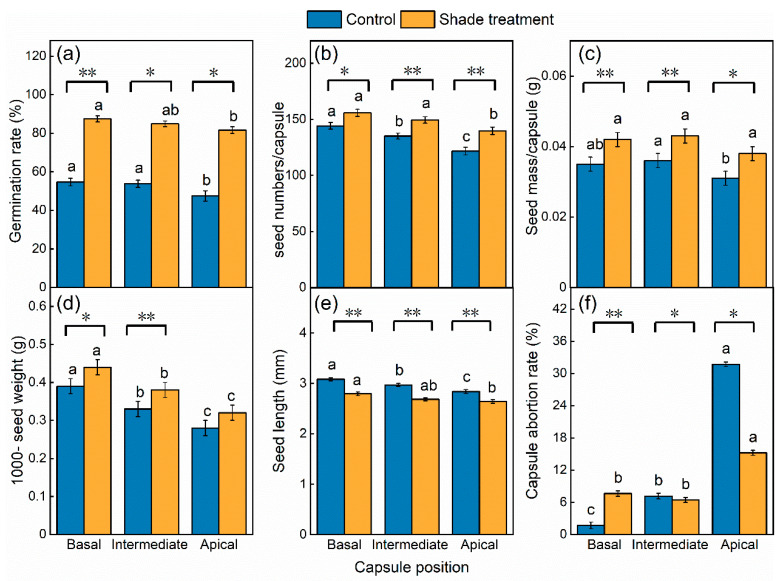
Effects of shade treatment and capsule position on the inflorescence on seed quality. (**a**) Germination rate. (**b**) Seed numbers per capsule. (**c**) Seed mass per capsule. (**d**) 1000-seed weight. (**e**) Seed length. (**f**) Capsule abortion rate. Different lowercase letters indicate a significant difference in capsule position (Basal, Intermediate, Apical) (*p* < 0.05). * indicates significant difference in shade treatment (shade treatment, Control) (* *p* < 0.05, ** *p* < 0.01). Blue color represents control treatment, orange color represents shading treatment.

Shade treatment and capsule position had significant effects on all seed quality traits (*p* < 0.05), while their interaction showed no effect except for capsule abortion rate (*p* < 0.001) (Table 2). Seed germination rate, seed mass per capsule, and 1000-seed weight increased significantly as the capsule position declined from apical to basal, regardless of control or shade treatment (*p* < 0.05) (Figure 3a,c,d), while capsule abortion rate decreased significantly (Figure 3f). Moreover, the positional effect was stronger under control than under shade. For example, the seed germination rate of the basal position was 7.13% higher than that of the top position under shade treatment, while it was 15.18% higher than the top position under control treatment. In contrast, the capsule abortion rate was 7.6% higher at the apical position than at the basal position under shade treatment, while it was 30% under control.

Shade significantly promoted all seed traits at different positions of the reproductive branch, except for seed length and capsule abortion rate (*p* < 0.001) (Figure 3a–d). Compared with the control, the average values of seed germination rate, seed numbers per capsule, and 1000-seed weight at three positions increased by 32.7%, 11.0%, and 15.2% under shade treatment, respectively. Regarding the different positions, the seed quality of the basal position was significantly higher than that of the intermediate and apical positions. For example, the seed germination rate, seed numbers, seed mass per capsule, and 1000-seed weight under shade treatment were 32.8%, 8%, 20%, and 12.8% higher than the control at the base position, while they were 34.2%, 14.8%, 22.6%, and 14.3% higher than the control at the apical position, respectively. In addition, the capsule abortion rate at different positions had a different sensitivity to light. For example, compared to control, shade significantly increased the capsule abortion rate by 5.95% at the basal position, but it was significantly decreased by 0.7% and 16.5% at the intermediate and apical positions, respectively.

### 2.3. Effects of Shade on Seed Yield and Yield Components

**Figure 4:**
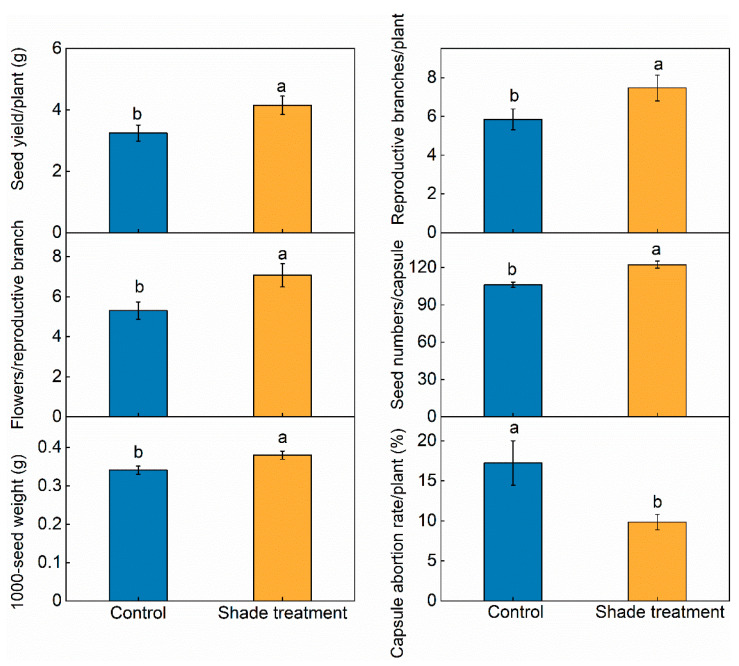
Effects of shading treatment on seed yield and yield components. Different lowercase letters indicate a significant difference in shade treatment. Blue color represents control treatment, orange color represents shading treatment (*p* < 0.05).

The shade treatment significantly increased seed yield and yield components (*p* < 0.05) (Figure 4). Seed yield, reproductive branches per plant, flowers per reproductive branch, seed numbers per capsule, and 1000-seed weight under shade treatment were 27.9%, 27.7%, 33.2%, 15.3%, and 11.4% higher than the control, respectively. However, the capsule abortion rate was 7.4% lower than that of the control.

**Figure 5:**
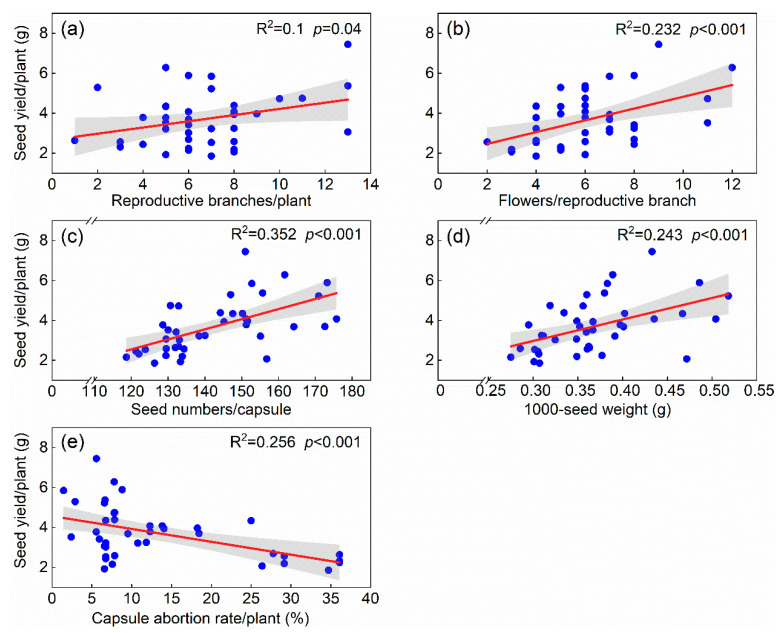
Relationships between seed yield and yield components. Shown are (**a**) Reproductive branches per plant. (**b**) Number of flowers per reproductive branch; (**c**) Number of seeds per capsule; (**d**) 1000-seed weight; and (**e**) Capsule abortion rate per plant. Each blue circle represents a sample point (*n* = 40). The red solid lines are significant regression lines, and shaded areas represent 95% confidence intervals.

**Figure 6:**
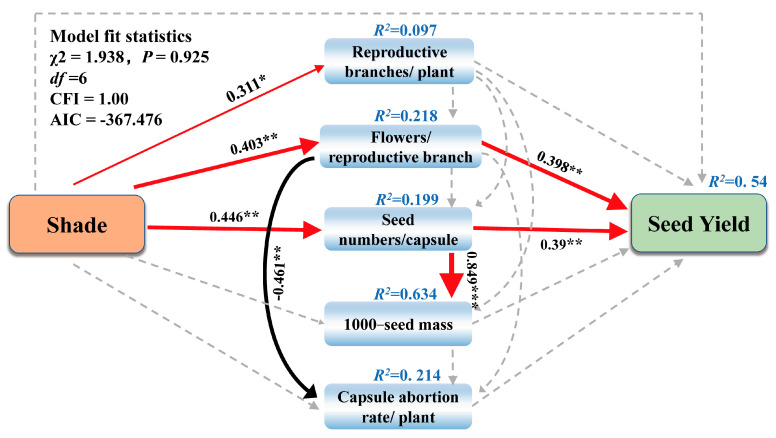
Structural equation modeling of shade on seed yield and yield components (*p* < 0.05). The structural equation model considered all plausible pathways through which shade treatments and yield components influencing seed yield. Red and black arrows represent significant positive and negative pathways, respectively, and gray dashed arrows indicate nonsignificant pathways. Bold numbers indicate the standard path coefficients. Arrow width is proportional to the strength of the relationship. *R*^2^ represent the proportion of variance explained for each dependent variable in the model. * *p* < 0.05, ** *p* < 0.01, *** *p* < 0.001.

Except for capsule abortion rate being significantly negatively correlated, all yield components were positively associated with seed yield (*p* < 0.05) (Figure 5). Structural equation modeling (SEM) partitioned the direct and indirect causes of association and measured the relative importance of each seed yield component under the shade treatment. The SEM adequately fitted the seed yield and yield components data (χ^2^ = 1.938, *df* = 6, *p* = 0.925; CFI = 1.00; standardized path coefficients are given in Figure 6). The final model explained 54% of the seed yield. It revealed that only flowers per reproductive branch and seed numbers per capsule had a direct effect on seed yield. The direct effect of flowers per reproductive branch on seed yield was 0.398, while the direct effect of seed numbers per capsule was 0.39 (Figure 6). Together, these two traits explained 54% of the variation in seed yield. In addition, there were two indirect pathways for the effect of shade treatment on seed yield: shade promoted flowers per reproductive branch and seed numbers per capsule, indirectly increasing seed yield.

## 3. Discussion

Shade significantly increased seed quality in our study, as evidenced by a significant increase in 1000-seed weight and germination rate. Variations in environment experienced by the maternal plant during seed development, the timing of seed maturity, and intrinsic hormone levels can cause differences in seed quality [25,26]. Our investigation of seed development found that shade did not advance or delay seed maturation, as the time of seed maturation was about 32 DAA regardless of control and shade treatments (Figure 2). It means that shading did not alter the course of seed development but might have affected the photosynthesis of plants and the distribution of photosynthetic products at later stages of development. As the quality of seeds at later stages of maturity was significantly different, this was demonstrated by the fact that 1000-seed weight, seed mass per capsule, seed germination rate, and germination index were significantly higher after 32 DAA in the shade treatment than in control (Figure 2). Excessive light exposure can lead to the overgeneration of reactive oxygen species [8], often resulting in the restriction of photosynthesis. Appropriate shading significantly increased the biomass accumulation of seedlings, encouraged flower bud differentiation, and promoted the accumulation of photosynthetic products [34]. Our findings determined that shade significantly promoted the number of reproductive branches per plant and the number of flowers per reproductive branch (Figure 6). Moderate shading during seed maturity promoted more carbohydrates produced by photosynthesis transferred to the seeds and an increase in 1000-seed weight and seed germination rate [35,36]. Our results demonstrated that plants under the control treatment exposed to stronger light developed fewer and lighter seeds; in contrast, the shade treatment alleviated this strong light inhibition, and the plants developed fuller seeds with higher germination rates and stronger reproductive potential. This is consistent with Bello et al. who found that the seed weight of velvetleaf increased with increasing the shade level [37]. In addition, shading of the mother plant promoted germination of soybean seeds [13].

In addition, it is clear from our results that the different positions on the reproductive branch had a significant effect on seed quality. Seeds located in the basal position had a significantly higher germination rate and 1000-seed weight than those in the apical positions. The cause of this discrepancy might be the physiologically older storage positions that inhibit the further development of younger ones [38]. Resource dominance by fruits nearer to the reproductive branches could lead to incomplete seed development in distal capsules. In essence, the number of successfully developed seeds in the distal capsule was reduced and the quality was lower, resulting in a small and lightweight seed area, which affected the reproductive potential. Moreover, many studies have shown that the size of seeds can be affected by plant size, plant density, the position on the parent plant, and resource availability [26,39]. In our study, the combined effects of shading and upper branch shading led to differences in the photosynthetic products received by seeds at different positions in plant, which resulted in more nutrient accumulation and more abundant seeds in the basal position. This was illustrated by the significant increase in seed mass per capsule and 1000-seed weight as capsule position declined from apical to base and the capsule abortion rate significant decreased in our results. This developmental pattern is common among taxa with different morphologies and life histories [38,40,41,42]. For example, the number of seeds in *Prunella vulgaris* (Labiatae) also decreases significantly with increasing position within the terminal raceme [43]. The average berry weight, the number of seeds per berry, and 100-seed weight of potatoes decreased from proximal to distal flower position [40]. This finding indicates that for *I. sinensis* var. *przewalskii,* an important quality strategy can be implemented by removing the apical inflorescence during early plant growth or increasing the planting density.

Environmental conditions prevailing during the growth period, especially canopy photosynthesis during flowering, and pod set are important determinants of seed yield [44,45,46]. Jiang and Egli reported that shade imposed from the first flower to the early pod fill reduced flower production and increased flower and pod abscission, resulting in reduced pod number and yield in soybean [47]. In contrast, our study indicated that shade significantly increased seed yield.

Shade significantly improved seed yield, which probably occurred because of the environmental change resulting from the shading. As mentioned above, insufficient seed filling due to excessive light in the capsule filling stage was alleviated by shading, allowing more photosynthetic products to be transferred to the seeds, increasing plant reproductive allocation and promoting more seed development and seed quality. For example, at 40 DAA of seed development, the number of seeds per capsule and seed mass per capsule increased by 10.2% and 50.3%, respectively, under shade treatment compared to the control. Clearly, shade can increase seed yield by promoting reproductive allocation. The increase in seed yield primarily resulted from the promotion of flowers per reproductive branch and seed numbers per capsule in our study. This was consistent with the findings that most seed yield variation was the direct effect of in pod and seed number as reported by many studies [48,49,50]. Similar results have been reported for meadow foam, where shade produced more flowers and seeds per square meter, and shade increased seed yield by 35% [51]. On the other hand, because of the racemose inflorescence of I. *sinensis* var. *przewalskii*, the capsule at the apical of the reproductive branch was exposed to excessive light, which resulted in a significant increase in the abortion rate of the apical capsule, while shade treatments and allocation and competition for resources promoted the development of flowers and capsules in the basal position, which significantly increased seed yield.

In addition, the temperature during plant growth also affected seed yield. As we know, exposure to temperatures above 30 °C causes reductions in plant growth and foliar damage, imposing severely impaired fitness on plants, reducing photosynthesis [52], inhibiting the growth of plants and the accumulation of photosynthetic products, and affecting the distribution of plant reproduction. Appropriate shading can reduce this adverse effect, promote flower bud differentiation and plant growth and development, as evidenced by shading significantly prompting more reproductive branches and the number of flowers per reproductive branch of plants, which in turn improved seed yield in our results.

Therefore, environmental conditions and management practices that favor the growth of flowers at the base of the reproductive branch may increase seed yield.

Overall, the strong positive correlation and substantial direct effect of flowers per reproductive branch and seed numbers per capsule on seed yield highlighted that these combined seed components are the most reliable indicators for selecting high seed yield varieties.

## 4. Materials and Methods

### 4.1. Plant Material, Growing Conditions, and Shade Treatments

*I. sinensis* var. *przewalskii* is a perennial herb of Bignoniaceae that is distributed in southwestern, northwestern to northeastern China under mingled forest (2000–2600 m above sea level). The flowering and fruiting season may last for nearly 5 months, from early June to early November [1]. The terminal racemes generally have 5–15 flowers, which are pale yellow and campanulate-shaped. The capsule is longhorn, slightly curved at the top, and contains many seeds of ovoid and irregular transparent wings on the edge.

Freshly matured seeds were collected on 20 September 2018 from a field on the Yuzhong Campus of Lanzhou University, Gansu Province, China. The capsules of at least 50 mature individual plants were hand collected and taken to the laboratory, where seeds were cleaned and dried at room temperature (RH 20–35%, 18–25 °C) for 1 week and then stored at 4 °C until used in experiments.

**Figure 7:**
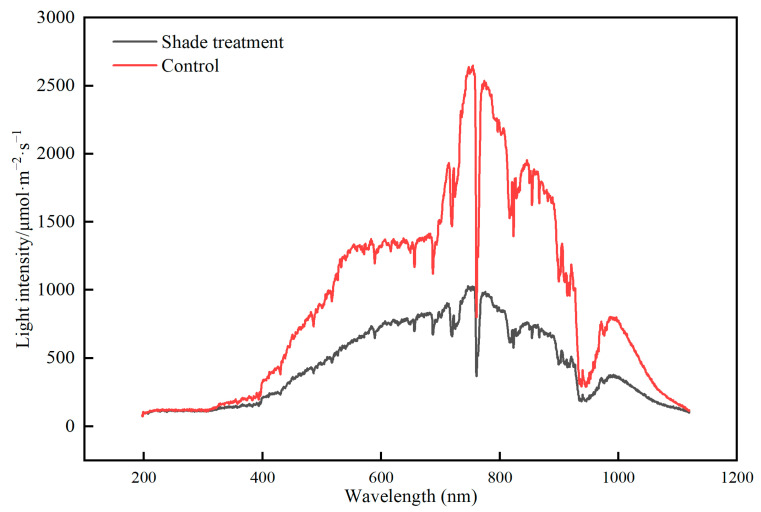
Light intensity under different shade treatments during the experiment.

Field experiments were conducted at the Yuzhong Campus of Lanzhou University in 2019 (104°16′ E, 35°94′ N). A randomized complete experimental design was applied with shade as treatment (shade, control) with three replications each, resulting in 6 plots. The shading treatment was consistently applied throughout the entire growth period, from planting to seed harvesting. Each plot was 3.5 m × 3 m. Twenty plants were planted in each plot with an individual distance of 30 cm × 24 cm in April 2019. The control treatment was to cover with white transparent PVC photosynthetic shed film (supported by 3.5 × 3 × 3 m; long × wide × high metal structures over the treated plots), the light transmittance of the film was 90%. Shading was achieved with two layers of black polyethylene fabric, the top and side halves were covered with shade cloth to provide a shading treatment, the light transmittance was 68%. This covering allowed free air movement in all directions to minimize possible differences in air temperature under the various screens. From planting to seed maturation, all plots were irrigated to minimize soil moisture stress. Irrigation was carried out once a week. The light intensity under different treatments during the experiments is shown in Figure 7.

### 4.2. Experimental Design

#### 4.2.1. Effects of Shade on Seed Development

To measure the growth and development characteristics of the capsule and seed, 120 fully open flowers were randomly tagged in each plot at full bloom (16 June 2019), random samples of 10 tagged capsules per plot were taken at 4-day intervals starting with the day the flowerings were tagged (4, 8, 12, 16, 20, 24, 28, 32, 36, and 40 days after anthesis (DAA)).

The samples were immediately taken to the laboratory, capsule length was measured as a straight line from peduncle to capsule tip, and seed numbers in each capsule were counted on fresh samples; shriveled or undeveloped seeds were not included. Fresh weight of the seeds was measured, and the seeds and capsule walls were oven-dried at room temperature (RH 20–35%, 18–25 °C) for 1 week for dry weight determination, 1000-seed weights were measured at the end of drying. The seeds in each capsule were tested for germination in an incubator at a temperature of 15–25 °C (12 h photoperiod), daily germination counts were carried out for 26 days, and their germination rate was recorded.

#### 4.2.2. Effects of Shade and Capsule Position on Seed Quality

At late seed maturity (15 August 2019), 30 reproductive branches with at least 5 well-developed capsules were meticulously chosen for each treatment (shade treatment, control) for the experiment. Capsules on reproductive branches were categorized as follows: 1–3 capsules closest to the lower end of the raceme were classified as “basal” capsules, 1–3 capsules at the uppermost part of the raceme were classified as “apical”, and capsules in the rest of the middle of the raceme were classified as “intermediate.” (If there were more than 9 flowers developed on the reproductive branch, the top 3 and bottom 3 were selected as the apical and basal, respectively, and the remaining flowers were used as the intermediate. If 6–9 flowers developed on the reproductive branches, the top 2 and bottom 2 flowers were selected as the apical and basal, respectively, and the remaining flowers were selected as the intermediate. If 5 flowers developed on the reproductive branch, one above and one below were selected as the apical and basal, respectively, and the remaining flowers were used as the intermediate).

Capsules were removed from different positions of the reproductive branches, and seeds were promptly removed from the capsules and left to dry at ambient temperature and low relative humidity for 2 weeks to determine the number, mass, and 1000-seed weight of seeds in each capsule. The length and area of seeds within each capsule were determined by multispectroscopy (VideometerLab, Videometer, Hørsholm, Denmark).

To evaluate the effect of different positions under two shading treatments of mature seeds, freshly collected seeds from all treatments of different positions (hereafter fresh seeds) were tested at 15/25 °C in light (12/12 h daily photoperiod, white fluorescent tubes, photon irradiance was 60 μmol m^−2^ s^−1^, 400–700 nm). For each position of each treatment, five replicates of 50 seeds were placed in 10 cm diameter Petri dishes on two sheets of filter paper (Shuangquan, Hangzhou, China) moistened with 7 mL distilled water, a total of 2 shading treatments ×* 3 position treatments were tested. Distilled water was added daily as needed to keep the filter paper moist. Germination of seeds incubated in light were monitored daily for at least 21 days until no further germination occurred for three consecutive days, and a seed was counted as germinated when the radicle was visible (≥2 mm). The germination percentage was scored as the number of germinated seeds/total number of live seeds in each dish. If the remaining seeds after stable germination were hard, they were considered viable, while soft, moldy seeds were considered non-viable. This experiment was performed in September 2019.

#### 4.2.3. Effects of Shade on Seed Yield and Yield Components

To measure seed yield and yield components, 10 plants were randomly selected from each plot, and the following traits were observed and/or calculated in full bloom: reproductive branches/plant, flowers/reproductive branch. And 50 capsules were randomly selected at seed maturity to measure seed numbers/capsule and calculate the capsule abortion rate/plant and seed mass per plant as seed yield (SY) at seed maturity.

### 4.3. Statistical Analysis

General Linear Models in the SPSS statistics were performed to investigate the effect on seed quality traits of two groups of variables and their interactions: (1) shading treatments and days after anthesis; (2) shading treatments and capsule position. In each model, these variables were used as fixed effects. Duncan’s test was used to compare means when significant differences were found. Before analysis, the percentage data were subjected to inverse sine transformation. Linear regressions were used to assess the relationship between yield and yield components, ANOVA was used to test whether the slopes of the linear regressions differed significantly among the treatments, and Origin 2018 was used for plotting all regression figures.

In addition, we constructed a structural equation model (SEM) of the interrelationships between shade treatments and yield, yield components, and abortion rate. We selected the optimal model by using path deletion and specification search methods and combining chi-square values (χ^2^, non-significant, *p* > 0.05), asymptotic residual mean square, comparative fitness index (CFI, whose value is generally greater than 0.9), and AIC (whose smaller value indicates better model fitness) [53]. Model construction and optimization were performed in R version 3.2.2 (R Foundation for Statistical Computing, Vienna, Austria, 2013), with the ‘lavaan’ package for SEM.

## 5. Conclusions

In brief, our study showed that shading treatment significantly improves seed quality and yield. Therefore, implementing shading practices in agricultural settings, such as increasing planting density, intercropping with taller shrubs or herbaceous plants to promote better growth and seed development, as well as removing part of the inflorescence at the top of the reproductive branches to enhance overall reproductive efficiency may be beneficial for increasing the seed yield and quality of *I. sinensis* var. *przewalskii*.

Further research and experimentation could explore different shading levels and techniques to identify the optimal conditions for maximizing seed yield and quality. Additionally, a comprehensive understanding of how various environmental factors besides shading influence seed development would be valuable in developing targeted cultivation strategies.

## Figures and Tables

**Table 1:** The effects of shading treatment, DAA, and their interaction on seed and capsule traits. using a general linear model (GLM). The asterisk refers to the interaction effect between variables before and after the asterisk.

Traits	Shade Treatment (ST)		Days after Anthesis (DAA)		ST×*DAA	
	F	*p*	F	*p*	F	*p*
Seed traits						
Germination rate	33.69	<0.01	55.52	<0.01	5.86	<0.01
Germination index	26.74	<0.01	40.73	<0.01	3.73	<0.01
Seed water content	12.28	<0.01	152.76	<0.01	1.81	0.08
1000-seed weight	29.34	<0.01	139.45	<0.01	7.10	<0.01
Seed numbers/capsule	11.15	<0.01	2.164	0.041	0.335	0.93
Seed mass/capsule	33.01	<0.01	33.53	<0.01	3.52	<0.01
Capsule traits						
Capsule dry weight	5.46	0.02	52.69	<0.01	0.20	0.99
Capsule length	7.18	0.01	15.17	<0.01	0.23	0.82

**Table 2:** The effects of shading treatment, capsule position, and their interaction on seed quality using a general linear model (GLM). The asterisk refers to the interaction effect between variables before and after the asterisk.

Traits	Shade Treatment (ST)		Capsule Position (CP)		ST×*CP	
	F	*p*	F	*p*	F	*p*
Germination rate	408.09	<0.001	5.59	0.007	0.49	0.616
Seed numbers/capsule	18.77	<0.001	6.69	<0.001	0.30	0.601
Seed mass/capsule	34.68	<0.001	3.67	0.027	0.06	0.939
1000-seed weight	41.76	<0.001	30.76	<0.001	0.81	0.446
Seed length	87.54	<0.001	15.41	<0.001	1.14	0.323
Capsule abortion rate	95.70	<0.001	1369.83	<0.001	122.36	<0.001

## References

[1] Chen S.T., Xing Y.W., Su T., Zhou Z.K., Dilcher E.D.L., Soltis D.E. (2012). Phylogeographic analysis reveals significant spatial genetic structure of *Incarvillea sinensisas* a product of mountain building. BMC Plant Biol..

[2] Chen S.T., Zhou Z.K., Guan K.Y., Nakata M. (2004). Karyomorphology of *Incarvillea* (*Bignoniaceae*) and its implications in distribution and taxonomy. Bot. J. Linn. Soc..

[3] Gao Y.P., Zhong G.Y., Shen Y.H. (2016). Chemical constituents from *Incarvillea sinensis* var. *przewalskii*. Chin. Tradit. Herbal Drugs.

[4] Li S., Chen H., Li C.Y., Wang M.W., Wei X.M., Chang Y.C. (2012). Orthogonal optimization of extraction process of total alkaloid in *Incarvillea sinensis* Lam. var. *przewalskii*. J. Gansu Univ. Chin. Med..

[5] Han C.J., Wang Q., Zhang H.B., Wang S.H., Song H.D., Hao J.M., Dong H.Z. (2018). Light shading improves the yield and quality of seed in oil-seed peony (*Paeonia ostii* Feng Dan). J. Integr. Agr..

[6] Cavagnaro J.B., Trione S.O. (2007). Physiological, morphological and biochemical responses to shade of *Trichloris crinita*, a forage grass from the arid zone of Argentina. J. Arid. Environ..

[7] Zhao D.Q., Hao Z.J., Tao J. (2012). Effects of shade on plant growth and flower quality in the herbaceous peony (*Paeonia lactiflora* Pall.). Plant Physiol. Bioch..

[8] Asada K. (2006). Production and scavenging of reactive oxygen species in chloroplasts and their functions. Plant Physiol..

[9] Laanisto L., Niinemets Ü. (2015). Polytolerance to abiotic stresses: How universal is the shade–drought tolerance trade-off in woody species?. Global Ecol. Biogeogr..

[10] Koller D. (1957). Germination-regulating mechanisms in some desert seeds, IV. *Atriplex dimorphostegia* Kar. et Kir. Ecology.

[11] Kollwr D., Roth N. (1964). Studies on the ecological and physiological significance of amphicarpy in *Gymnarrhena micrantha* (Compositae). Am. J. Bot..

[12] Shinomura T., Nagatani A., Hanzawa H., Kubota M., Watanabe M., Furuya M. (1996). Action spectra for phytochrome A-and B-specific photoinduction of seed germination in *Arabidopsis thaliana*. Proc. Natl. Acad. Sci. USA.

[13] Chen F., Zhou W.G., Yin H., Luo X.F., Chen W., Liu X., Wang X., Meng Y., Feng L., Qin Y. (2020). Shading of the mother plant during seed development promotes subsequent seed germination in soybean. J. Exp. Bot..

[14] Datta S.C., Evenari M., Gutterman Y. (1970). The heteroblasty of *Aegilops ovata*. Israel J. Bot..

[15] Gray D., Thomas T.H. (1982). Seed germination and seedling emergence as influenced by the position of development of the seed on, and chemical applications to, the parent plant. Physiology and Biochemistry of Seed Development, Dormancy and Germination.

[16] Rylski I., Spigelman M. (1986). Effect of shading on plant development, yield and fruit quality of sweet pepper grown under conditions of high temperature and radlation. Sci. Hortic..

[17] Steiman S., Idol T., Bittenbender H.C., Gautz L. (2011). Shade coffee in Hawai‘i—Exploring some aspects of quality, growth, yield, and nutrition. Sci. Hortic..

[18] Board J.E., Harville B.G. (1996). Growth dynamics during the vegetative period affects yield of narrow-row, late-planted soybean. Agron. J..

[19] Myers R.L., Brun W.A., Brenner M.L. (1987). Effect of raceme-localized supplemental light on soybean reproductive abscission. Crop Sci..

[20] Schou J.B., Jeffers D.L., Streeter J.G. (1978). Effects of reflectors, black boards, or shades applied at different stages of plant development on yield of soybeans. Crop Sci..

[21] Mathew J.P., Herbert S.J., Zhang S.H., Rautenkranz A.A.F., Litchfield G.V. (2000). Differential response of soybean yield components to the timing of light enrichment. Agron. J..

[22] Han C.J., Wang Q., Zhang H.B., Dong H.Z. (2019). Seed development and nutrient accumulation as affected by light shading in oilseed peony (*Paeonia ostii* ‘Feng Dan’). Sci. Hortic..

[23] Marin M., Blandino C., Laverack G., Toorop P., Powell A.A. (2019). Responses of *Primula vulgaris* to light quality in the maternal and germination environments. Plant Biology.

[24] Baskin C.C., Baskin J.M. (1998). Seeds: Ecology, Biogeography, and, Evolution of Dormancy and Germination.

[25] Gray D., Steckel J.R.A. (1985). Parsnip (*Pastinaca sativa*) seed production: Effects of seed crop plant density, seed position on the mother plant, harvest date and method, and seed grading on embryo and seed size and seedling performance. Ann. Appl. Biol..

[26] Hendrix S.D. (1984). Variation in seed weight and its effects on germination in *Pastinaca sativa* L. (*Umbelliferae*). Am. J. Bot..

[27] Koller D. (1970). Analysis of the dual action of white light on germination of *Atriplex dimorphostegia* (*Chenopodiaceae*). Israel J. Bot..

[28] Keiller D.R., Morgan D.J. (1988). Distribution of ^14^carbon-labelled assimilates in flowering plants of oilseed rape (*Brassica napus* L.). J. Agr. Sci..

[29] Evans E.J. (1984). Pre-anthesis growth and its influence on seed yield in winter oilseed rape. Aspects Appl. Biol..

[30] Inanaga S., Kumura A., Etho K., Tsunoda K.J. (1986). Studies on matter production of rape plants: VIII. Effects of shading, leaf cutting and pod thinning treatments on yield and its components. Jpn. J. Crop Sci..

[31] Tayo T.O., Morgan D.G. (1979). Factors influencing flower and pod development in oil-seed rape (*Brassica napus* L.). J. Agr. Sci..

[32] Svečnjak Z., Varga B., Butorac J. (2006). Yield components of apical and subapical ear contributing to the grain yield responses of prolific maize at high and low plant populations. J. Agron. Crop Sci..

[33] Habekotté B. (1993). Quantitative analysis of pod formation, seed set and seed filling in winter oilseed rape (*Brassica napus* L.) under field conditions. Field Crop Res..

[34] Imru N.O., Wogderess M.D., Gidada T.V. (2015). A study of the effects of shade on growth, production and quality of coffee (*Coffea arabica*) in Ethiopia. Int. J. Agric. Stat. Sci..

[35] Doust J.L., Doust L.L. (1988). Plant Reproductive Ecology: Patterns and Strategies.

[36] Kohri K., Saitoh K., Kuroda T., Kumano S. (1998). Significance of Flower Differentiation and Development in the Process of Determining Soybean Yield: Effects of shading treatment on the number of floral buds and pod sets. Jpn. J. Crop Sci..

[37] Bello I.A., Owen M.D.K., Hatterman-Valentp H.M. (1995). Effect of shade on velvetleaf (*Abutilon theophrasti*) growth, seed production, and dormancy. Weed Technol..

[38] Katepa-Mupondwa F.M., Smith J.S.R., Barnes D.K. (1996). Influence of parent and temperature during pollination on alfalfa seed weight and number of seeds per pod. Can. J. Plant Sci..

[39] Wulff R.D. (1986). Seed size variation in *Desmodium paniculatum*: I. Factors affecting seed size. J. Ecol..

[40] Almekinders C.J.M., Neuteboom J.H., Struik P.C. (1995). Relation between berry weight, number of seeds per berry and 100-seed weight in potato inflorescences. Sci. Hortic..

[41] Georgieva N. (2020). Seed Heterogeneity in Dependence of Their Position on the Mother Plant in *Lupinus albus* L.. Banats J. Biotechnol..

[42] Liu B., Zhou X.M., Qu D.N. (2012). Relationship among seed size from different seed positions at several-seeded pod in soybean. J. Food Agric. Environ..

[43] Diggle P.K. (1995). Architectural effects and the interpretation of patterns of fruit and seed development. Annu. Rev. Ecol. Syst..

[44] Board J.E., Harville B.G. (1992). Explanations for greater light interception in narrow-vs. wide-row. Crop Sci..

[45] Taylor H.W., Mason W.K., Bennie A.T.P., Rowse H.R. (1982). Responses of soybeans to two row spacings and two soil water levels. I. An analysis of biomass accumulation, canopy development, solar radiation interception and components of seed yield. Field Crop Res..

[46] Willcott J., Herbert S.J., Liu Z.Y. (1984). Leaf area display and light interception in short-season soybeans. Field Crop Res..

[47] Jiang H., Egli D. (1993). Shade induced changes in flower and pod number and flower and fruit abscission in soybean. Agron. J..

[48] Iannucci A., Di Fonzo N., Martiniello P. (2002). Alfalfa (*Medicago sativa* L.) seed yield and quality under different forage management systems and irrigation treatments in a Mediterranean environment. Field Crop Res..

[49] Sengul S. (2006). Using path analysis to determine lucerne (*Medicago sativa* L.) seed yield and its components. N. Z. J. Agr. Res..

[50] Sharma K., Walia N. (1996). Growth and Yield of Soybean *Glycine max* (L.) Merill as Influenced by Light Intensity and Cytokinin. Environ. Ecol..

[51] Norberg O.S., Fiez T.E., Jolliff G.D., Seddigh M., Crane J.M. (1993). Shading and Crop-Cover Effects on Meadowfoam Oil Yield. Agron. J..

[52] Lippmann R., Babben S., Menger A., Delker C., Quint M. (2019). Development of wild and cultivated plants under global warming conditions. Curr. Biol..

[53] Grace J.B. (2006). Structural Equation Modeling and Natural Systems.

